# Impact of Different Epoxidation Approaches of Tall Oil Fatty Acids on Rigid Polyurethane Foam Thermal Insulation

**DOI:** 10.3390/ma14040894

**Published:** 2021-02-13

**Authors:** Arnis Abolins, Ralfs Pomilovskis, Edgars Vanags, Inese Mierina, Slawomir Michalowski, Anda Fridrihsone, Mikelis Kirpluks

**Affiliations:** 1 Polymer Laboratory, Latvian State Institute of Wood Chemistry, Dzerbenes St. 27, LV-1006 Riga, Latvia; arnisaabolins@gmail.com (A.A.); ralfs.pomilovskis@gmail.com (R.P.); edgars.vanags6@gmail.com (E.V.); anda.fridrihsone@edi.lv (A.F.); 2 Institute of Technology of Organic Chemistry, Faculty of Materials Science and Applied Chemistry, Riga Technical University, P. Valdena 3/7 St., LV-1048 Riga, Latvia; inese.mierina@rtu.lv; 3 Department of Chemistry and Technology of Polymers, Cracow University of Technology, Warszawska 24, 31-155 Cracow, Poland; slawomir.michalowski@pk.edu.pl

**Keywords:** tall oil fatty acids, ion-exchange resin, lipase enzyme catalyst, high functionality polyols, rigid polyurethane foam

## Abstract

A second-generation bio-based feedstock—tall oil fatty acids—was epoxidised via two pathways. Oxirane rings were introduced into the fatty acid carbon backbone using a heterogeneous epoxidation catalyst-ion exchange resin Amberlite IR-120 H or enzyme catalyst *Candida antarctica* lipase B under the trade name Novozym^®^ 435. High functionality bio-polyols were synthesised from the obtained epoxidated tall oil fatty acids by oxirane ring-opening and subsequent esterification reactions with different polyfunctional alcohols: trimethylolpropane and triethanolamine. The synthesised epoxidised tall oil fatty acids (ETOFA) were studied by proton nuclear magnetic resonance. The chemical structure of obtained polyols was studied by Fourier-transform infrared spectroscopy and size exclusion chromatography. Average molecular weight and polydispersity of polyols were determined from size exclusion chromatography data. The obtained polyols were used to develop rigid polyurethane (PU) foam thermal insulation material with an approximate density of 40 kg/m^3^. Thermal conductivity, apparent density and compression strength of the rigid PU foams were determined. The rigid PU foams obtained from polyols synthesised using Novozym^®^ 435 catalyst had superior properties in comparison to rigid PU foams obtained from polyols synthesised using Amberlite IR-120 H. The developed rigid PU foams had an excellent thermal conductivity of 21.2–25.9 mW/(m·K).

## 1. Introduction

Due to environmental concerns, new regulatory policies and a shift in consumer requirements, renewable resources for polymer materials have been widely studied in the last decade [1]. In the past, as well as nowadays, fatty acids present in plants in the form of triglycerides are one of the most appropriate raw products for the manufacturing of bio-based materials [2]. Various chemical modification methods, such as epoxidation and ring-opening, hydroformylation, transesterification, ozonolysis, amidation and thiol-ene coupling [3], have been used to obtain various monomers and polymers with low toxicity, convenient availability and relatively low price [4,5,6,7]. Epoxides are one of the most versatile intermediates to be further used for the synthesis of different compounds and has a wide commercial use because of its high reactivity [8].

Numerous epoxidation methods of different kind of vegetable oils, such as canola-rapeseed [9,10], palm [11], cottonseed [12], soybean [13], castor [14], linseed [15], mahua [16] and grape seed [17] among others, have been reported. In addition, non-edible plant oils, such as tall oil [18,19,20] and jatropha [21,22], have been successfully epoxidised previously. However, the use of vegetable oil for industrial uses is in direct competition with food and feed production. As mentioned, there are sources of plant-derived fatty acids that do not compete, such as tall oil [23,24].

Tall oil fatty acids (TOFA) are important renewable feedstock, which is obtained as a side stream from the softwood Kraft pulping process. After fractional distillation of crude tall oil, a more pure form of TOFA is obtained containing at least 97% free fatty acids (mainly a mixture of 48–52% of oleic and 43–48% of linoleic acid) and less than 3% other components, such as rosin acids and unsaponifiables [25,26,27,28]. The relatively high level of unsaturation makes TOFA a suitable raw material for the introduction of reactive functional group using the unsaturated hydrocarbon C=C double bonds, making them suitable for further processing into polymers [29].

The most widely used epoxidation method is the well-known Prilezhaev reaction where peracid is reacted with olefins [20,30,31,32]. Peracids are conventionally formed in situ from hydrogen peroxide and short-chain carboxylic acid in the presence of highly acidic catalysts [32,33]. However, during epoxidation, acidic catalysts, such as sulphuric acid and acidic ion exchange resins, and carboxyl acids as oxygen carriers, such as formic or acetic acid, exacerbate undesirable side product formation through oxirane rings [18,20,34]. Moreover, the higher the temperature of the epoxidation reaction, the greater the frequency of side reactions [20]. Studies previously carried out by our group showed that if free fatty acids containing unprotected carboxyl groups, as they are in TOFA, are epoxidised, the use of acidic components has even more significance for side reactions occurrence [18,20]. To avoid the formation of side products, other epoxidation routes have to be explored.

Some studies indicate that chemo-enzymatic epoxidation, where acidic catalysts are replaced by lipase, overall is a milder route to free fatty acid conversion into epoxides [19] than the well-known Prilezhaev rection. The advantages of chemo-enzymatic epoxidation are lower reaction temperature [35], the absence of acidic catalysts [36,37] and even lipase reusability [36], which if all taken in to account can result in substantially higher oxirane ring introduction into the substrate. In addition, lipases are highly selective to limit the frequency of side reactions [38]. Moreover, it is possible to prevent the use of additional oxygen carriers if free fatty acids are chemo-enzymatically epoxidised. Lipases can turn free fatty acids into highly reactive peroxy fatty acids, which subsequently epoxidise unsaturated bonds [39], thus improving the feasibility of reaction as there are no acidic catalysts and additional oxygen carriers needed. However, different factors, such as solvent, temperature, pH and presence of activators or deactivators etc., can influence the activity of lipases [40].

After epoxidation, one of the most easily obtainable functional groups are hydroxyl groups, which are essential for polyurethane (PU) production. PUs are a class of polymers that are commonly used in a wide variety of applications to produce high-performance materials. The primary uses for PUs are flexible and rigid foams, sealants, elastomers, adhesives, and coatings [30,41,42,43,44,45,46,47]. Usually, PUs are obtained by polycondensation reaction between isocyanates and hydroxyl group containing compounds [48,49,50]. Hydroxyl group compounds can be polyols with low, medium or high functionality with low, medium or high hydroxyl values (OH value), respectively. Polyols with high average hydroxyl group functionality are needed for the production of rigid PU foams to ensure high dimensional, mechanical and thermal stability of the material [51]. A combination of epoxy ring-opening and transesterification or transamidation of fatty acids with polyfunctional alcohols can lead to such polyols, which would contain primary OH groups to ensure high cross-link density of obtained PU polymer matrix [52,53,54].

The goal of this study was to compare two different TOFA epoxidation catalysts—ion exchange resin Amberlite IR-120 H and enzymatic catalyst *Candida antarctica* lipase B with a trade name Novozym^®^ 435—and their influence on the properties of resulting polyols and rigid PU foams. In this study, a second-generation bio-based feedstock—TOFA—was epoxidised via two pathways resulting in two different epoxidised tall oil fatty acids (ETOFA). Afterwards, two different polyols were developed using the two different ETOFA and employing oxirane ring-opening and subsequent esterification reactions with two different polyfunctional alcohols (trimethylolpropane (TMP) and triethanolamine (TEOA)). The four developed polyols were used to obtain rigid PU foam thermal insulation material. Its common characteristics, such as thermal conductivity, apparent density and compression strength, were analysed and compared.

## 2. Materials and Methods

### 2.1. Materials

TOFA (trade name “FOR2”) with a high content of fatty acids (>96%), low content of rosin acids (1.9%) and unsaponifiables (1.8%) was ordered from Forchem Oyj (Rauma, Finland). Glacial acetic acid (AcOH), puriss, ≥99.8%; hydrogen peroxide (H_2_O_2_), purum p.a., ≥35%; acetanhydride, puriss, ≥99%; 4-(dimethylamino)pyridine (DMAP), reagent plus, ≥99%; N,N-dimethylformamide (DMF), ACS reagent, ≥99.8%, water content ≤150 ppm; potassium hydroxide, puriss, ≥85%; potassium iodide, ACS reagent, ≥99%; tetraethylammonium bromide, reagent grade, 98%; perchloric acid, ACS reagent, 70%; dichloromethane, puriss p.a., ACS reagent; anhydrous sodium sulphate, puriss; TMP, reagent grade, 97%, were ordered from Sigma-Aldrich (Schnelldorf, Germany). Amberlite IR-120 H, strongly acidic, hydrogen form and sodium thiosulphate fixanals 0.1 M and Hanus solution, volumetric 0.1 M IBr were ordered from Fluka (Steinheim, Germany). Lipase Novozym^®^ 435 (immobilised on acrylic resin) was kindly supplied by Novozymes A/S (Bagsvaerd, Denmark). Tetrafluoroboric acid solution, 48 wt.% in H_2_O (HBF_4_), was ordered from Alfa Aesar (Kandel, Germany). TEOA, 99.2%, was ordered from Huntsman (Rotterdam, The Netherlands), and was used as purchased.

For the development of rigid PU foams, the following materials were used as purchased: two tertiary amine-based catalysts Polycat^®^ 5, Polycat^®^ NP10 as well as 30 wt.% of potassium acetate in diethylene glycol (PC CAT TKA 30) (Air Products and Chemicals Inc., Halfweg, The Netherlands); Niax Silicone L-6915 as a surfactant (Momentive Performance Materials Inc., Rotterdam, Germany); tris (1-chloro-2-propyl phosphate 99% (TCPP) as a flame retardant (Albermarle, Louvain-la-Neuve, Belgium)) and cyclopentane as a physical blowing agent (Sigma-Aldrich, Schnelldorf, Germany). Desmodur 44V20 L was purchased from (Covestro, Krefeld, Germany), and was used as the isocyanate component for all PU materials. It is a solvent-free product based on 4,4′-diphenylmethane diisocyanate (pMDI) and contains oligomers of high functionality. The average functionality is 2.8–2.9 and the isocyanate group (–NCO) content of 30.5–32.5 wt.%.

### 2.2. Epoxidation of TOFA with Ion Exchange Resin Amberlite IR-120 H

The epoxidation of TOFA was carried out in a four-necked round bottom flask. A thermocouple, mechanical stirrer, dropping funnel and a reflux condenser were attached to the necks of the flask. The epoxidation of TOFA was achieved by in-situ generated peroxyacetic acid, which forms from acetic acid and hydrogen peroxide in the presence of an acidic catalyst. During the epoxidation, the molar ratio of TOFA (double bonds-155 g I_2_/100 g) to H_2_O_2_ and AcOH was 1.0:1.5:0.5. At first, the calculated amount of TOFA (700.0 g), acetic acid (128.5 g) and ion exchange resin Amberlite IR-120 H (140.0 g, 20 wt.% of TOFA weight), as the catalyst, was added to the flask. The flask was immersed in a thermostatic water bath (preheated to 40 °C). The speed of the mechanical stirrer was set to 600 rpm, and the mixture was started to stir. A hydrogen peroxide/water (35%/65%) solution (638.5 g) was poured into a dropping funnel. When the content of the flask reached 40 °C, hydrogen peroxide solution was added dropwise to the round bottom flask in a time interval of 30 min. Meanwhile, the temperature of the reaction medium was slowly increased to 60 °C. After the complete addition of hydrogen peroxide, the reaction medium was continued to stir for 6 h at 60 °C and 600 rpm [18]. Afterwards, the reaction mixture was poured into a separating funnel and washed four times with warm (T = 60 °C) distilled water. The product was dried using a rotatory vacuum evaporator to remove water residues. As a result, ETOFA were obtained exhibiting the acid value of 144 mg KOH/g, oxirane content of 2.30 mmol/g and iodine value of 27.0 g I_2_/100 g and characterised by pomegranate red colour. An acronym ETOFA_IR is used for the ETOFA synthesised via TOFA epoxidation with ion exchange resin Amberlite IR-120 H.

### 2.3. Epoxidation of TOFA with Novozym^®^ 435

The epoxidation of TOFA was carried out in a four-necked round bottom flask. The flask was immersed in a water bath and equipped with a mechanical stirrer, a reflux condenser, a thermocouple and a dropping funnel. Using the data that was obtained from the previous study [55], the optimal epoxidation parameters were determined and used for TOFA epoxidation.

During the epoxidation, the molar ratio of double bond present in TOFA and H_2_O_2_ was 1.0:2.0. The required amount of TOFA (700.0 g) was poured in the flask, and necessary amount of *Candida antarctica* lipase B (Novozym^®^ 435) (22.4 g, 3.2 wt.% of TOFA weight) was added. The mixture of TOFA and catalyst was heated to 44 ± 0.1 °C. Afterwards, the necessary amount of 32% H_2_O_2_ water solution (1038.2 g) was added to the reactants dropwise through the dropping funnel. The rate of addition speed was adjusted so that the whole peroxide was added within 30 min. After the complete addition of hydrogen peroxide, the reaction medium was continued to stir for 5.5 h at 44 ± 0.1 °C and 500 rpm. Afterwards, the reaction mixture was poured into a separating funnel and washed four times with warm (T = 60 °C) distilled water. The product was dried using a rotatory vacuum evaporator to remove water residues. As a result, ETOFA were obtained exhibiting the acid value of 146 mg KOH/g, oxirane content of 3.28 mmol/g and iodine value of 17.0 g I_2_/100 g and characterised by pomegranate red colour. An acronym ETOFA_E is used for the ETOFA obtained from TOFA epoxidation with lipase catalyst Novozym^®^ 435.

### 2.4. Synthesis of Polyols Using Two Different ETOFA

**Figure 1:**
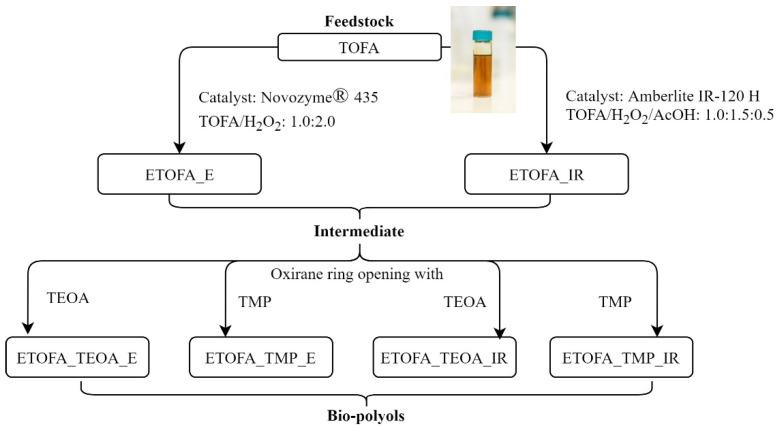
Scheme of developed tall oil fatty acids (TOFA)-based bio-polyols and intermediates.

High functionality polyols were synthesised by functionalising ETOFA_IR or ETOFA_E, which were obtained by epoxidising TOFA using two epoxidation catalysts either Amberlite IR-120 H or Novozym^®^ 435. Bio-based polyols were synthesised by opening the oxirane ring of ETOFA_IR or ETOFA_E and subsequent esterification with various polyfunctional alcohols, such as TMP and TEOA. For bio-polyols synthesised from intermediate ETOFA_IR, the following acronyms were used ETOFA_TMP_IR and ETOFA_TEOA_IR depending on the used polyfunctional alcohol for epoxide ring-opening. For polyols obtained from TOFA epoxidation with lipase catalyst Novozym^®^ 435, the following acronyms were used ETOFA_TMP_E and ETOFA_TEOA_E. The general scheme of bio-polyol development is depicted in Figure 1.

Due to the two different chemical processes that are carried out simultaneously, namely, oxirane ring-opening reaction and esterification reaction, the molar amount of the polyfunctional alcohol needed for polyol synthesis is calculated using the following equation:

n_MP_ = n_KG_ + n_EG_, (1)

where n_MP_ is the molar amount of the polyfunctional alcohol used for oxirane ring-opening and esterification reaction (TMP or TEOA), in mol; n_KG_ is the molar amount of the ETOFA carboxylic groups, in mol and n_EG_ is the molar amount of the ETOFA oxirane groups, in mol.

**Figure 2:**
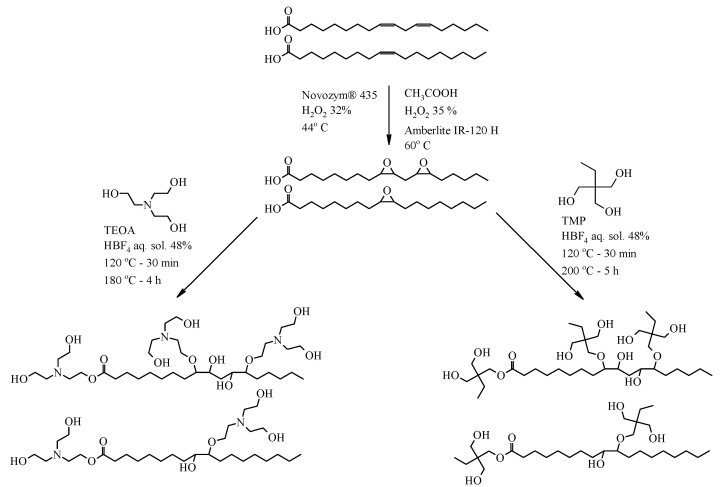
Idealised chemical structure of developed bio-polyols.

To obtain TOFA-based bio-polyols, the oxirane ring-opening was first carried out in the four-necked round bottom flask. The multifunctional alcohol/amine and tetrafluoroboric acid solution, 48 wt.% in H_2_O and as a catalyst, 0.4 wt.% of ETOFA mass, was added, see Table 1 for corresponding mass for each type of polyols. The flask was immersed into an oil thermobath, and a mechanical stirrer was inserted into the central neck. A purge gas tube, Liebig condenser, and a dropping funnel were attached to the vacant necks. The mixer was set to 400 rpm, and the flow of purge gas (argon) through the flask was provided, while the content of the flask was heated up to 120 °C. When the required temperature of 120 °C for oxirane ring-opening was reached, 200 g of ETOFA were added dropwise to the flask in a time interval of 20 min. After the complete addition of ETOFA, the reaction medium was continued to stir for 30 min at the temperature of 120 °C to open the oxirane rings completely. Afterwards, the synthesis temperature was increased to carry out the esterification reactions. The ETOFA_TMP_IR and ETOFA_TMP_E polyol synthesis were carried out at 200 °C, whereas ETOFA_TEOA_IR and ETOFA_TEOA_E synthesis were carried out at 180 °C (Figure 2). The stirring of the reaction medium and the argon gas flow was retained until the acid value of the product decreased below 5 mg KOH/g [54]. After which, the synthesis was considered to be finished, and the bio-polyol was obtained for further rigid PU foam development.

### 2.5. Characterisation of Products and Precursors

The obtained bio-polyols were characterised by hydroxyl and acid values calculated according to ISO 4629-2:2016 and ISO 2114:2000 testing standards using titrimetric methods. The epoxy content was calculated in accordance with ASTM D1652-04:2004. The viscosity of polyols was measured at 25 °C using the Thermo Science HAAKE (Medium-High Range Rotational Viscometer, Thermo Fisher Scientific, Waltham, MA, USA). Polyol density was determined using a series of hydrometers. In a thermostatic bath at 20 °C, a graduated cylinder filled with polyol was immersed for 20 min, and afterwards, the density was measured. The moisture content was measured using the Denver Instrument Model 275KF automatic titrator (Denver Instrument, Bohemia, NY, USA) using Karl Fisher titration.

Polyol structure was analysed using Fourier-transform infrared spectrometry data (FTIR), which were obtained with a Thermo Scientific Nicolet iS50 spectrometer (Thermo Fisher Scientific, Waltham, MA, USA) at a resolution of 4 cm^−1^ (32 scans). The FTIR data were collected using attenuated total reflectance technique with ZnSe and diamond crystals. Moreover, ^1^H NMR spectra for the samples were recorded on a Bruker spectrometer (Bruker BioSpin AG, Fällanden, Switzerland) at 500 MHz. The chemical shifts (δ) are reported in ppm. The residual chloroform peak was used as an internal reference (δ = 7.26 ppm). Size exclusion chromatography from Knauer equipped with refractive index detector (Detector RI) and polystyrene/divinylbenzene matrix gel column with a measurement range up to 30,000 Da at tetrahydrofuran (THF) eluent flow of 1.0 mL/min was used to analyse the number-average molecular weight (M_n_) and number-average functionality (f_n_) of the synthesised bio-polyols. The polyols f_n_ was calculated based on hydroxyl values and M_n_ as seen from Equation (2) [52].

f_n_ = M_n_ × OH_value_ × 56,110^−1^, (2)

where f_n_ is the number-average functionality, OH groups/mol; M_n_ is the number-average molecular weight, g/mol; OH_valuel_ is the hydroxyl value of the polyol, mg KOH/g and 56,100 is equivalent weight of KOH, in milligrams.

### 2.6. Rigid PU foam Preparation and Characterisation

For rigid PU foam development, ETOFA-based bio-polyols were used. For the PU foams obtained from bio-based polyols synthesised from ETOFA using Amberlite IR-120 H and polyfunctional alcohols like TMP and TEOA, the following acronyms PU_TMP_IR and PU_TEOA_IR were used. Similarly, for the PU foams obtained from bio-based polyols synthesised from ETOFA using Novozym^®^ 435 and polyfunctional alcohols like TMP and TEOA, the following acronyms PU_TMP_E and PU_TEOA_E were used. In Table 2, the developed rigid PU foam formulations are depicted. In order to obtain rigid PU foams using newly synthesised bio-polyols a previously synthesised bio-polyol with lower functionality based on tall oil (TO) esterification with TEOA was also used (TO_TEOA with OH value of 334 mg KOH/g, the water content of 0.45 wt.%, the viscosity of 280 mPa·s at 25 °C, f_n_ = 2.4 and M_n_ = 391 g/mol). The polyol component was obtained by weighing all the required components presented in Table 2 (polyols, blowing agent, flame retardant, catalysts and surfactant) and stirring them with a mechanical stirrer at 2000 rpm for 1 min. The polyol system was then conditioned in a sealed container at room temperature for at least 2 h to de-gas the mixed air, and afterwards, PU foams were prepared. The isocyanate index was chosen to be 150 for all PU foams. To produce rigid PU foams, isocyanate (pMDI) and a polyol portion were weighed and mixed at 2000 rpm for 15 s with a mechanical stirrer. After that, the reactive mixture was poured into an open-top mould [54].

The content of renewable materials was determined based on the mass of renewable materials used in the formulation of the PU foams. The rigid PU foam formulations were designed to obtain foams with an apparent density of ~40 kg/m^3^. The physical and mechanical properties of the foams were measured in accordance with the following standards: foam density—ISO 845:2009, closed cell content—ISO 4590:2003, compression strength—ISO 844:2009 and thermal conductivity—ISO 8301:1991. The compression strength of the PU foams was tested parallel and perpendicular to foam rise with one offset from ISO 844:2009 standard—sample size; cylinders with a diameter of 20 mm and a height of 22 mm were tested. The mechanical testing of PU foams was done using Zwick/Roell 1000 N testing machines (Zwick Roell Group, Ulm, Germany)

## 3. Results and Discussion

### 3.1. Characteristics of Synthesised High Functionality Polyols

The key characteristics of synthesised bio-polyols, such as OH value, acid value, moisture content, viscosity, density, f_n_, M_n_ and polydispersity (p_d_) are summarised in Table 3.

The synthesised TOFA bio-polyols have high OH values and low acid value, which is typical and necessary for polyols to be used for rigid PU foam production. Unfortunately, the bio-polyols that were synthesised using TMP reached very high viscosity. It can be explained by the intermolecular hydrogen bonding of the polyol chemical structure as well as the higher molecular weight of the polyols. The f_n_ of synthesised bio-polyols is high as it ranges from 8.1 to almost 12.6. Polyols with such high f_n_ are rarely used as the only polyol in rigid PU foam formulation. Therefore, such polyols’ main application is their use as cross-linking reagents in rigid PU foam development.

### 3.2. FTIR Analysis of Synthesised Bio-Polyols from ETOFA

**Figure 3:**
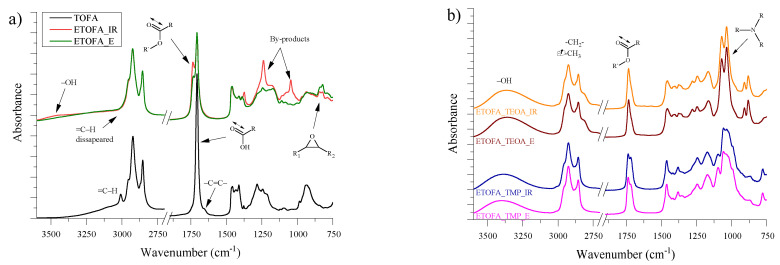
FTIR spectra: (**a**) spectra of TOFA, epoxidised tall oil fatty acids synthesised via TOFA epoxidation with ion exchange resin Amberlite IR-120 H (ETOFA_IR) and ETOFA obtained from TOFA epoxidation with lipase catalyst Novozym^®^ 435 (ETOFA_E) and (**b**) spectrum of the resulting TOFA-based bio-polyols.

The FTIR spectra of TOFA, ETOFA_IR and ETOFA_E are depicted in Figure 3a.

The oxirane ring stretching vibration peak appeared at 823 cm^−1^ after epoxidation of TOFA. Furthermore, for ETOFA_E, its intensity is higher when compared with ETOFA_IR, which correlates with the determined oxirane content from titrimetric analysis data of 3.28 and 2.30 mmol/g, respectively. The introduction of oxirane rings coincides with the disappearance of =C–H and –C=C– absorption peaks at 3009 and 1659 cm^−1^, respectively. The side reactions of oxirane ring cleavage during TOFA epoxidation occurred for both epoxidation methods and are confirmed by the occurrence of a C=O ester stretching shoulder peak for the both ETOFA intermediate products at 1736 cm^−1^. In case of ETOFA_IR, its intensity is higher as the oxirane ring cleavage side reactions could occur with COOH groups of TOFA as well as acetic acid present in reaction medium among other reactants. The side reaction occurrence was also confirmed by the appearance of the broad –OH stretching vibration peak between 3600 and 3100 cm^−1^, which for ETOFA_IR was more intensive than ETOFA_E. In the case of ETOFA_IR, the C–O bond absorption peaks at 1240 and 1046 cm^−1^ of various by-products were also more distinguishable. It can be observed that the Novozym^®^ 435 is more selective epoxidation catalyst, and ETOFA_E could be better suited for polyol development.

When TOFA-based bio-polyols were obtained (Figure 3b), the oxirane ring stretching vibration peak at 823 cm^−1^ disappeared. This correlates with a titrimetric analysis of oxirane content in all TOFA-based bio-polyols, which was lower than 0.01 mmol/g. These kinds of changes in FTIR spectra confirm conversion towards the desired bio-polyol products. A very noticeable distinction between bio-polyols synthesised from TMP and TEOA is the C–N stretching peak of a tertiary amine group that appeared at ~1036 cm^−1^ when oxirane ring was opened with TEOA. The tertiary amine group introduction in chemical structure could provide the developed bio-polyol with autocatalytic properties when rigid PU foam is developed. For all four polyols, the carboxylic group C=O stretching vibration at 1707 cm^−1^ of TOFA shifted towards C=O stretching ester vibration at ~1733 cm^−1^. For all feedstock, TOFA and intermediates, both ETOFAs and four obtained polyols a typical symmetric and asymmetric –CH_2_– stretching peaks were observed at ~2930 and 2860 cm^−1^. The broad absorption peak between 3600 and 3100 cm^−1^ of all spectra of synthesised bio-polyols was identified as characteristic stretching vibrations of the O–H group. The intensity of peak between 3600 and 3100 cm^−1^ of synthesised bio-polyols correlated with the increase in OH value obtained from the titrimetric analysis of the polyols presented in Table 3.

### 3.3. NMR Analysis of Epoxidated TOFA Using Different Catalysts

**Figure 4:**
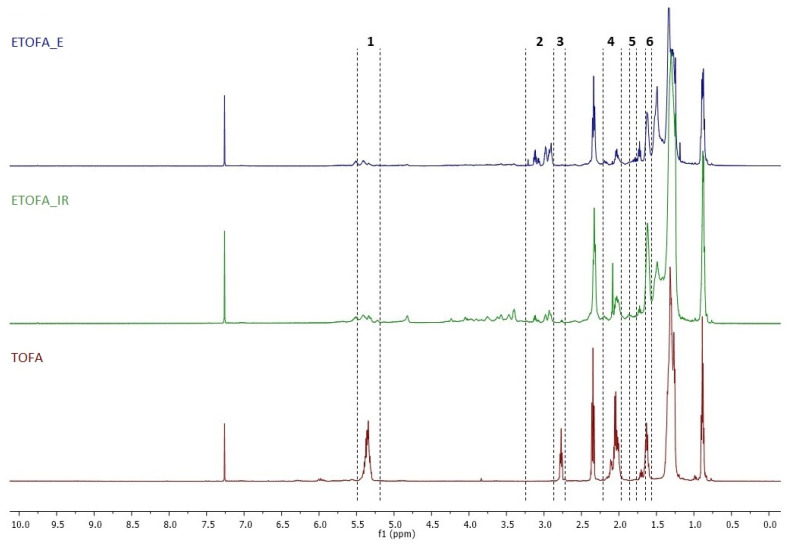
^1^H-NMR of TOFA and TOFA-catalysed epoxidation by ion-exchange resin (ETOFA_IR) and TOFA-catalysed epoxidation by lipase catalyst Novozym^®^ 435 (ETOFA_E) (500 MHz, CDCl_3_). Abbreviations: 1—protons of double bond; 2—protons of oxirane moiety; 3—protons of *bis*-allylic position; 4—protons of allylic position; 5—CH_2_ protons of oxiran-2-yl moiety; 6—CH_2_ protons of *bis-*(oxiran-2-yl) moiety.

Tall oil mainly consists of linoleic acid and oleic acid. According to ^1^H NMR spectra, the ratio of both these fatty acids is about 1:1: characteristic signal for linoleic acid is *bis*-allylic protons at 2.8 ppm (Figure 4). The amount of oleic acid was calculated from the amount of allylic protons at 1.9–2.1 ppm.

^1^H NMR spectra demonstrate that enzyme-catalysed reaction leads to a rather clean formation of epoxides (characteristic signals at 2.9–3.1 ppm), without cleavage of the oxirane ring in ETOFA_E product. The ETOFA_E contained up to 60% of epoxide moieties (calculated per number of double bonds in the starting material) and nearly 20% of untreated double bond moieties (signals at 5.1–5.7 ppm).

The yield of oxiranes was significantly smaller when ion exchange resin was used as the epoxidation catalyst. Although the oxirane moiety was formed, a significant amount of oxirane cleavage products is observed (characteristic signals in the range of 3.3–4.5 ppm). According to the ^1^H NMR spectra, the product contained less than 20% of epoxide moieties (calculated per number of double bonds). Besides, the conversion of double bond was not full (signals at 5.1–5.7 ppm).

### 3.4. SEC Analysis of Synthesised High Functionality Bio-Polyols

**Figure 5:**
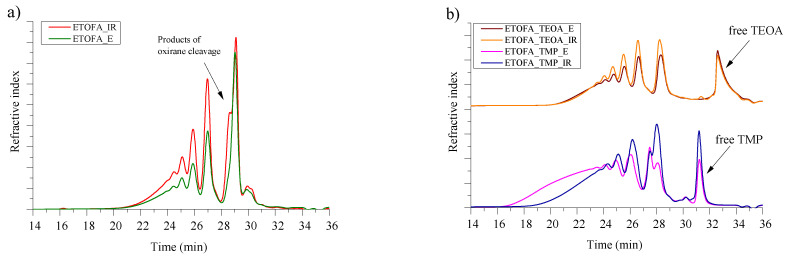
SEC chromatograms of (**a**) different ETOFA and (**b**) developed TOFA bio-polyols.

The SEC chromatograms of different ETOFA and synthesised bio-polyols are depicted in Figure 5. The oxirane cleavage products discussed in previous paragraphs were also visible in SEC chromatogram of ETOFA_IR as a shoulder of epoxidised fatty acid products at the retention time of ~29 min. The oxirane ring-opening dimerisation, trimerisation and oligomerisation products were identified at retention times of ~27, 26 and 25 min, respectively, in ETOFA of both types of epoxidation methods. Although, the higher selectivity of Novozym^®^ 435 catalyst ensured a lower amount of dimerisation products.

The obtained polyols had relatively similar characteristics when compared by used oxirane opening polyfunctional amine/alcohol despite ETOFA_E having fewer products of oxirane cleavage side reactions. The SEC chromatograms of ETOFA_TEOA_E and ETOFA_TEOA_IR are almost the same. Unfortunately, in the case of ETOFA_TMP_E polyol, an even larger amount of oligomerisation products has been identified. The oligomerisation of ETOFA_TMP_E polyol led to increased M_n_ of 2112 g/mol compared to ETOFA_TMP_IR polyol with M_n_ of 1279 g/mol (See Table 3). The oligomerisation could be explained due to the lower reactivity of TMP towards oxirane ring-opening. The method of oxirane ring-opening has to be further optimised to reduce the amount of oligomerisation products as they increase the viscosity of the bio-polyols, which is already considerable. High viscosity is undesirable, as it complicates the industrial upscale of the technology and production of rigid PU foams. Moreover, peaks of unreacted TEOA and TMP have been identified at the retention time of ~33 and 31 min, respectively. The presence of unreacted polyfunctional amines/alcohols is undesired but not critical, as they contain OH groups and can react with isocyanate in the formation of PU material. Obtained bio-polyols are a mixture of different TOFA derivatives with high OH group functionality and are suitable for rigid PU foam development.

### 3.5. Characteristics of Rigid PU Foams Developed from Synthesised High Functional Bio-Polyols

The main goal of the study was to obtain good-quality rigid PU foams using two different types of bio-polyols obtained from ETOFA_IR and ETOFA_E and to compare their common physical and mechanical properties. Synthesised TOFA-based bio-polyols were used to prepare rigid PU foams by formulations shown in Table 2. Typical characteristics of obtained rigid PU foams are summarised in Table 4.

**Figure 6:**
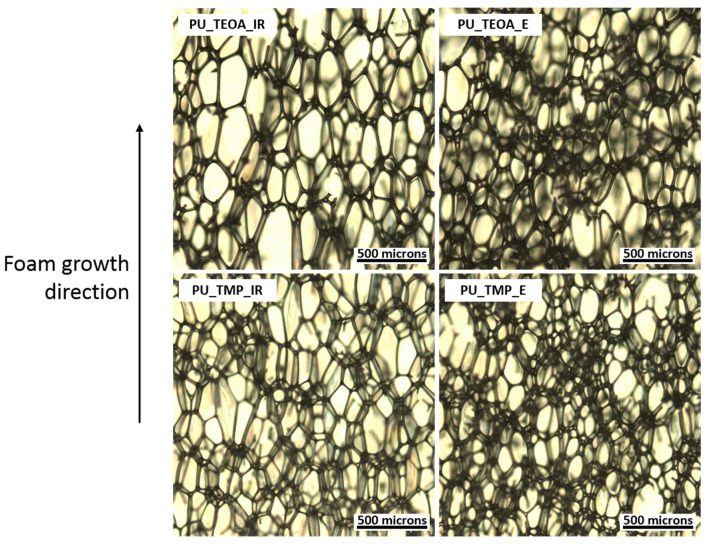
Microscope images of developed rigid polyurethane (PU) foams.

The foaming start time of rigid PU foam formulations developed from TEOA-type bio-polyols is on par with formulations from TMP-type bio-polyol at ~30 s. The presence of tertiary amine groups in TEOA-type bio-polyols expresses autocatalytic properties. Thus, this allowed to bypass the use of Polycat^®^ 5 catalyst. This could be beneficial because Polycat^®^ 5 is an additive catalyst with low boiling temperature, and it could leak out from the foamed material over time. This is not desirable as amine catalysts tend to have a strong odour which could influence the air quality in a rigid PU foam application environment, such as inside of buildings. The TEOA-type bio-polyol autocatalytic activity also influenced a faster foaming rise time of 61 s when compared to the foaming rise time of ~70 s of TMP-type bio-polyol formulations. The apparent density of developed rigid PU foams was almost the same and varied between 35.4 and 38.5 kg/m^3^ range. The slightly higher apparent density of PU_TMP_E foam can be explained by higher viscosity of ETOFA_TMP_E polyol. Nevertheless, the closed cell content of all samples was 90% or above it, which is typical for rigid PU foams intended for thermal insulation application. The thermal conductivity for all developed rigid PU foams was quite low, reaching values between 21.2 and 23.3 mW/(m·K), which is considered appropriate for industrial use. The type of ETOFA used for polyols synthesis did not influence the thermal conductivity of obtained rigid PU foams. The thermal conductivity of rigid PU foams mostly depends on the gas composition inside the material’s closed cells. The slight difference in the cell size (see Figure 6) of developed rigid PU foams did not influence the thermal conductivity.

**Figure 7:**
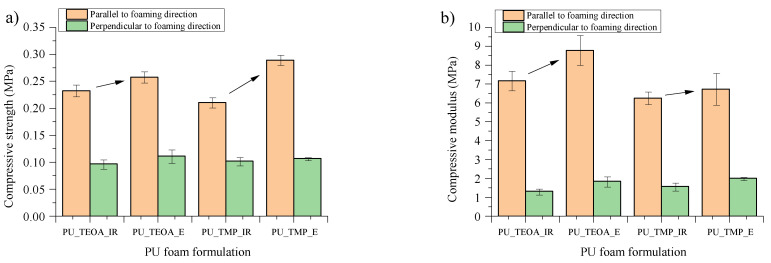
Compression properties (**a**) compressive strength and (**b**) compressive modulus of developed rigid PU foams normalised to the average apparent density of 40 kg/m^3^.

The compression strength and compression modulus of ETOFA_TEOA_IR-, ETOFA_TEOA_E-, ETOFA_TMP_IR- and ETOFA_TMP_E-based rigid PU foams were measured in parallel and perpendicular to foaming direction and are depicted in Figure 7. The data have been normalised to the average apparent density of 40 kg/m^3^, according to Hawkins et al. [56], to compare the mechanical properties. Developed rigid PU foams had significant anisotropicity as the mechanical properties varied depending on the testing direction. Anisotropicity is typical for rigid PU foams that are obtained in open-type mould. Rigid PU foam cells tend to elongate with the foaming direction, as seen in Figure 6. Nevertheless, the compression strength was ~0.2 MPa, which is typical for this type of material applied for civil engineering [57].

A slight difference can be distinguished when the mechanical properties are compared regarding the used epoxidation method (ETOFA_IR and ETOFA_E). Rigid PU foams obtained from ETOFA_E showed a slight increase in compression properties due to the higher functionality of the derived polyols compared to polyols obtained from ETOFA_IR. A higher functionality of bio-polyol will result in the higher cross-link density of the obtained PU polymer matrix, which will increase the mechanical properties of rigid PU foam. In addition, the increase in mechanical properties can also be explained by slightly smaller cell size rigid PU foams obtained from ETOFA_E as depicted in Figure 5.

The common properties of the developed rigid PU foams were compared to other rigid PU foams that were obtained using other bio-based polyols (Table 5). The rigid PU foams used for comparison were produced from different bio-based polyols, such as polyols from waste cooking oil (PU 60 PWCO) [30], pumpkin seed oil (PU-PSO) [47], soybean oil-derived polyol (PU-0) [46] as well as commercially used polyether type polyols with the trade name Rokopol^®^ RF-551 (PU-BMC/0) [45] and Raypol^®^ 4218 [47]. The chosen rigid PU foams from literature data were selected because they had somewhat similar apparent density with closed cell structure. Some differences in-between foams can be distinguished, namely, the isocyanate index, which was 110, whereas the present study describes rigid PU foams with the isocyanate index of 150. Nevertheless, a comparison can be made to assess developed materials suitability as a thermal insulation. The compression properties of TOFA-based rigid PU foams were similar to materials described in the most recent literature; the slightly lower values can be explained by the lower apparent density. The thermal conductivity between the compared rigid PU foams is similar. In the case of TOFA-based foams, it is even slightly better than in most of the compared foams. It is crucial to mention that the TOFA-based rigid PU foams were developed using only bio-based polyols in the formulation. The developed TOFA-based high functionality polyols can be used as a cross-linking reagent and might be used as a replacement for sorbitol-based polyether type polyols.

## 4. Conclusions

Two different epoxidation catalyst have been applied for tall oil fatty acid epoxidation and the obtained epoxidised oil have been used for high functionality bio-polyol synthesis. Application of lipase-based catalyst allowed to obtain epoxidised tall oil fatty acids with a lower amount of side reaction by-products and higher content of oxirane rings when compared to oil epoxidated with ion exchange resin type catalyst (3.28 and 2.30 mmol/g, respectively). The higher oxirane content of epoxidised oil yielded bio-polyols with higher number average functionality. Unfortunately, a significant amount of oligomerisation products formation was observed during bio-polyol synthesis, which increased the viscosity of bio-polyols. To decrease the amount of oligomerisation products, the bio-polyol synthesis process has to be further optimised. The synthesised bio-polyols were used to obtain rigid PU foams. The type of chosen epoxidation route, ion-exchange resin or enzyme catalyst, for tall oil fatty acid epoxidation did not significantly influence the properties of foams. Only mechanical properties had a slight increase for rigid PU foams obtained from feedstock synthesised via chemo-enzymatic epoxidation due to the higher number average functionality of bio-polyols. Overall, developed rigid PU foams had typical properties as for material applied as thermal insulation in civil engineering.

## Figures and Tables

**Table 1:** Multifunctional alcohols/amines used in the bio-polyol syntheses.

Multifunctional Alcohol for Polyol Synthesis	Mass (g) Added if Polyol is Synthesised from ETOFA_IR	Mass (g) Added if Polyol is Synthesised from ETOFA_E
TEOA	145.0	175.4
TMP	130.4	157.8

**Table 2:** Polyurethane (PU) foam formulations and renewable material content in PU foams.

Components		Reagents, Parts by Weight			
		PU_TEOA_IR	PU_TEOA_E	PU_TMP_IR	PU_TMP_E
Bio-polyols	ETOFA_TEOA_IR	85.0	–	–	–
	ETOFA_TEOA_E	–	85.0	–	–
	ETOFA_TMP_IR	–	–	85.0	–
	ETOFA_TMP_E	–	–	–	85.0
	TO_TEOA	10.0	10.0	10.0	10.0
	Glycerol	5.0	5.0	5.0	5.0
Blowing agents	c-pentane	12.0	12.0	12.0	10.0
	Water	2.0	2.0	2.0	2.0
Catalysts	Polycat^®^ 5	–	–	0.5	0.5
	Polycat^®^ NP10	3.0	3.0	3.0	3.0
	PC CAT TKA 30	1.5	1.5	1.5	1.5
Surfactant	L-6915	2.5	2.5	2.5	2.5
Flame retardant	TCPP	31 (8 wt.%)	30 (8 wt.%)	32 (8 wt.%)	27 (8 wt.%)
Isocyanate	pMDI	243.4	218.3	246.2	190.5
Isocyanate index	–	150	150	150	150
Renewable materials in PU foam, %		16.3	18.1	16.2	19.8

**Table 3:** Common characteristics of developed tall oil fatty acids (TOFA)-based bio-polyols.

Polyol	OH Value, mg KOH/g	Acid Value, mg KOH/g	Moisture, %	Viscosity (20 °C), mPa·s	Density (20 °C), g/cm^3^	f_n_	M_n_, g/mol	p_d_
ETOFA_TEOA_IR	510	<5	0.14	7400	1.047	8.1	893	1.78
ETOFA_TEOA_E	519	<5	0.05	10,800	1.048	8.3	899	1.88
ETOFA_TMP_IR	427	<5	0.24	77,000	1.056	9.7	1279	1.58
ETOFA_TMP_E	335	<5	0.06	278,300	1.058	12.6	2112	1.57

**Table 4:** The common characteristic of developed rigid PU foams based on TOFA-type bio-polyols.

PU Material	PU_TEOA_IR	PU_TEOA_E	PU_TMP_IR	PU_TMP_E
Technological parameters	–			
Foaming start time, s	28	29	32	23
Foaming rise time, s	61	61	78	67
PU foam apparent density, kg/m^3^	36.4	35.4	35.8	38.5
Closed cell content, %	90	91	91	95
Thermal conductivity, mW/(m·K)	23.0	23.3	21.3	21.2

**Table 5:** The common characteristic of developed rigid PU foams comparison with other materials.

PU Material	Compressive Strength, MPa	Compressive Modulus, MPa,	PU Foam Apparent Density, kg/m^3^	Closed Cell Content, %	Thermal Conductivity, mW/(m·K)
PU_TEOA_IR	0.191	5.6	36.4	90.0	23.0
PU_TEOA_E	0.195	6.7	35.4	91.0	23.3
PU_TMP_IR	0.198	5.9	35.8	91.0	21.3
PU_TMP_E	0.309	7.2	38.5	95.0	21.2
PU_60_PWCO ^1^	0.275	n.a	41.3	85.3	26.4
PU-BMG/0 ^2^	0.248	n.a	42.1	87.5	22.5
PU-0 ^3^	0.250	6.4	37.0	88.0	n.a
PU-PSO ^4^	0.238	n.a	40.8	n.a	33.9
Raypol^®^ 4218 ^4^	0.303	n.a	44.1	n.a	36.8

^1^ Data were taken from M. Kurańska et al. [30]; ^2^ Data were taken from M. Barczewski et al. [45]; ^3^ Data were taken from S. Czlonka et al. [46]; ^4^ Data were taken from P. Ekkaphan et al. [47].

## Data Availability

Data Sharing is not applicable.

## Abbreviations

ETOFA	Epoxidised tall oil fatty acids
ETOFA_E	Epoxidised tall oil fatty acids obtained from tall oil fatty acids epoxidation with lipase catalyst Novozym^®^ 435
ETOFA_IR	Epoxidised tall oil fatty acids obtained from tall oil fatty acids epoxidation with ion exchange resin Amberlite IR-120 H
ETOFA_TEOA_E	Polyol that is obtained from epoxidised tall oil, enzyme is used as a catalyst, oxirane ring opened with triethanolamine
ETOFA_TEOA_IR	Polyol that is obtained from epoxidised tall oil, ion exchange resin is used as a catalyst, oxirane ring opened with triethanolamine
ETOFA_TMP_E	Polyol that is obtained from epoxidised tall oil if enzyme is used as a catalyst, oxirane ring opened with trimethylolpropane
ETOFA_TMP_IR	Polyol that is obtained from epoxidised tall oil if ion exchange resin is used as a catalyst, oxirane ring opened with trimethylolpropane
–NCO	Isocyanate group
OH value	Hydroxyl value
pMDI	4,4′-diphenylmethane diisocyanate
PU	Polyurethane
PU_TEOA_E	Polyurethane formulated using polyol that is obtained from epoxidised tall oil, enzyme is used as a catalyst, oxirane ring opened with triethanolamine
PU_TEOA_IR	Polyurethane formulated using polyol that is obtained from epoxidised tall oil, ion exchange resin is used as a catalyst, oxirane ring opened with triethanolamine
PU_TMP_E	Polyurethane formulated using polyol that is obtained from epoxidised tall oil, enzyme is used as a catalyst, oxirane ring opened with trimethylolpropane
PU_TMP_IR	Polyurethane formulated using polyol that is obtained from epoxidised tall oil, ion exchange resin is used as a catalyst, oxirane ring opened with trimethylolpropane
TEOA	Triethanolamine
TMP	Trimethylolpropane
TO_TEOA	Polyol that is obtained by tall oil tall oil esterification with trietanolamine
TOFA	Tall oil fatty acids

## References

[1] Zhang C., Garrison T.F., Madbouly S.A., Kessler M.R. (2017). Recent advances in vegetable oil-based polymers and their composites. Prog. Polym. Sci..

[2] Biermann U., Bornscheuer U., Meier M.A.R., Metzger J.O., Schäfer H.J. (2011). Oils and Fats as Renewable Raw Materials in Chemistry. Angew. Chem. Int. Ed..

[3] Desroches M., Escouvois M., Auvergne R., Caillol S., Boutevin B. (2012). From vegetable oils to polyurethanes: Synthetic routes to polyols and main industrial products. Polym. Rev..

[4] Li Y., Luo X., Hu S. (2015). Bio-Based Polyols and Polyurethanes.

[5] Montero De Espinosa L., Meier M.A.R.R. (2011). Plant oils: The perfect renewable resource for polymer science?!. Eur. Polym. J..

[6] Lomège J., Negrell C., Robin J.J., Lapinte V., Caillol S. (2019). Oleic acid-based poly(alkyl methacrylate) as bio-based viscosity control additive for mineral and vegetable oils. Polym. Eng. Sci..

[7] Noreen A., Zia K.M., Zuber M., Tabasum S., Zahoor A.F. (2016). Bio-based polyurethane: An efficient and environment friendly coating systems: A review. Prog. Org. Coat..

[8] Tan S.G., Chow W.S. (2010). Biobased epoxidized vegetable oils and its greener epoxy blends: A review. Polym. Plast. Technol. Eng..

[9] Mungroo R., Pradhan N.C., Goud V.V., Dalai A.K. (2008). Epoxidation of canola oil with hydrogen peroxide catalyzed by acidic ion exchange resin. J. Am. Oil Chem. Soc..

[10] Kirpluks M., Kalnbunde D., Walterova Z., Cabulis U. (2017). Rapeseed Oil as Feedstock for High Functionality Polyol Synthesis. J. Renew. Mater..

[11] Lee P.L., Wan Yunus W.M.Z., Yeong S.K., Abdullah D.K., Lim W.H. (2009). Optimization of the epoxidation of methyl ester of palm fatty acid distillate. J. Oil Palm Res..

[12] Dinda S., Patwardhan A.V., Goud V.V., Pradhan N.C. (2008). Epoxidation of cottonseed oil by aqueous hydrogen peroxide catalysed by liquid inorganic acids. Bioresour. Technol..

[13] Patel M.M., Patel B.P., Patel N.K. (2012). Utilization of soya-based polyol for High solid PU-coating application. Int. J. Plast. Technol..

[14] Sinadinović-Fišer S., Janković M., Borota O. (2012). Epoxidation of castor oil with peracetic acid formed in situ in the presence of an ion exchange resin. Chem. Eng. Process. Process Intensif..

[15] Abolins A., Yakushin V., Vilsone D. (2018). Properties of polyurethane coatings based on linseed oil phosphate ester polyol. J. Renew. Mater..

[16] Goud V.V., Patwardhan A.V., Pradhan N.C. (2006). Studies on the epoxidation of mahua oil (Madhumica indica) by hydrogen peroxide. Bioresour. Technol..

[17] de Haro J.C., Izarra I., Rodríguez J.F., Pérez Á., Carmona M. (2016). Modelling the epoxidation reaction of grape seed oil by peracetic acid. J. Clean. Prod..

[18] Abolins A., Kirpluks M., Vanags E., Fridrihsone A., Cabulis U. (2020). Tall Oil Fatty Acid Epoxidation Using Homogenous and Heterogeneous Phase Catalysts. J. Polym. Environ..

[19] Kirpluks M., Vanags E., Abolins A., Fridrihsone A., Cabulis U. (2019). Chemo-enzymatic oxidation of tall oil fatty acids as a precursor for further polyol production. J. Clean. Prod..

[20] Vanags E., Kirpluks M., Cabulis U., Walterova Z. (2018). Highly functional polyol synthesis from epoxidized tall oil fatty acids. J. Renew. Mater..

[21] Goud V.V., Patwardhan A.V., Dinda S., Pradhan N.C. (2007). Kinetics of epoxidation of jatropha oil with peroxyacetic and peroxyformic acid catalysed by acidic ion exchange resin. Chem. Eng. Sci..

[22] Hazmi A.S.A., Aung M.M., Abdullah L.C., Salleh M.Z., Mahmood M.H. (2013). Producing Jatropha oil-based polyol via epoxidation and ring opening. Ind. Crops Prod..

[23] Maisonneuve L., Chollet G., Grau E., Cramail H. (2016). Vegetable oils: A source of polyols for polyurethane materials. OCL.

[24] Hachemi I., Kumar N., Mäki-Arvela P., Roine J., Peurla M., Hemming J., Salonen J., Murzin D.Y. (2017). Sulfur-free Ni catalyst for production of green diesel by hydrodeoxygenation. J. Catal..

[25] Chemistry C. (2016). Preperation of biodiesel and separation of hemicellulose. Cellul. Chem. Technol..

[26] Aro T., Fatehi P. (2017). Tall oil production from black liquor: Challenges and opportunities. Sep. Purif. Technol..

[27] Panda H. (2013). Handbook on Tall Oil Rosin Production, Processing and Utilization.

[28] Demirbas A. (2011). Methylation of wood fatty and resin acids for production of biodiesel. Fuel.

[29] Lubguban A.A., Ruda R.J.G., Aquiatan R.H., Paclijan S. (2017). Soy-Based Polyols and Polyurethanes. KIMIKA.

[30] Kurańska M., Leszczyńska M., Kubacka J., Prociak A., Ryszkowska J. (2020). Effects of Modified Used Cooking Oil on Structure and Properties of Closed-Cell Polyurethane foams. J. Polym. Environ..

[31] Omonov T.S., Kharraz E., Curtis J.M. (2016). The epoxidation of canola oil and its derivatives. RSC Adv..

[32] Ranganathan S., Sieber V. (2017). Development of semi-continuous chemo-enzymatic terpene epoxidation: Combination of anthraquinone autooxidation and the lipase-mediated epoxidation process. React. Chem. Eng..

[33] Re R.N., Proessdorf J.C., La Clair J.J., Subileau M., Burkart M.D. (2019). Tailoring chemoenzymatic oxidation: Via in situ peracids. Org. Biomol. Chem..

[34] Cai X., Zheng J.L., Aguilera A.F., Vernières-Hassimi L., Tolvanen P., Salmi T., Leveneur S. (2018). Influence of ring-opening reactions on the kinetics of cottonseed oil epoxidation. Int. J. Chem. Kinet..

[35] Zhang X., Wan X., Cao H., Dewil R., Deng L., Wang F., Tan T., Nie K. (2017). Chemo-enzymatic epoxidation of Sapindus mukurossi fatty acids catalyzed with Candida sp. 99–125 lipase in a solvent-free system. Ind. Crops Prod..

[36] Mashhadi F., Habibi A., Varmira K. (2018). Determination of Activation Energy and Ping-Pong Kinetic Model Constants of Enzyme-Catalyzed Self-Epoxidation of Free Fatty Acids using Micro-reactor. Catal. Lett..

[37] Danov S.M., Kazantsev O.A., Esipovich A.L., Belousov A.S., Rogozhin A.E., Kanakov E.A. (2017). Recent advances in the field of selective epoxidation of vegetable oils and their derivatives: A review and perspective. Catal. Sci. Technol..

[38] Kumar A., Dhar K., Kanwar S.S., Arora P.K. (2016). Lipase catalysis in organic solvents: Advantages and applications. Biol. Proced. Online.

[39] Milchert E., Malarczyk-Matusiak K., Musik M. (2016). Technological aspects of vegetable oils epoxidation in the presence of ion exchange resins: A review. Polish J. Chem. Technol..

[40] Mateo C., Palomo J.M., Fernandez-Lorente G., Guisan J.M., Fernandez-Lafuente R. (2007). Improvement of enzyme activity, stability and selectivity via immobilization techniques. Enzyme Microb. Technol..

[41] Ivdre A., Soto G.D., Cabulis U. (2016). Polyols Based on Poly(ethylene terephthalate) and Tall Oil: Perspectives for Synthesis and Production of Rigid Polyurethane Foams. J. Renew. Mater..

[42] Zhang C., Ding R., Kessler M.R. (2014). Reduction of epoxidized vegetable oils: A novel method to prepare bio-based polyols for polyurethanes. Macromol. Rapid Commun..

[43] Zeltins V., Yakushin V., Cabulis U., Kirpluks M. (2017). Crude Tall Oil as Raw Material for Rigid Polyurethane Foams with Low Water Absorption. Solid State Phenom..

[44] Pfister D.P., Xia Y., Larock R.C. (2011). Recent advances in vegetable oil-based polyurethanes. ChemSusChem.

[45] Barczewski M., Kurańska M., Sałasińska K., Michałowski S., Prociak A., Uram K., Lewandowski K. (2020). Rigid polyurethane foams modified with thermoset polyester-glass fiber composite waste. Polym. Test..

[46] Członka S., Strakowska A., Strzelec K., Kairyte A., Kremensas A. (2020). Bio-based polyurethane composite foams with improved mechanical, thermal, and antibacterial properties. Materials.

[47] Ekkaphan P., Sooksai S., Chantarasiri N., Petsom A. (2016). Bio-Based Polyols from Seed Oils for Water-Blown Rigid Polyurethane Foam Preparation. Int. J. Polym. Sci..

[48] Alagi P., Ghorpade R., Jang J.H., Patil C., Jirimali H., Gite V., Hong S.C. (2018). Functional soybean oil-based polyols as sustainable feedstocks for polyurethane coatings. Ind. Crops Prod..

[49] Omrani I., Farhadian A., Babanejad N., Shendi H.K., Ahmadi A., Nabid M.R. (2016). Synthesis of novel high primary hydroxyl functionality polyol from sunflower oil using thiol-yne reaction and their application in polyurethane coating. Eur. Polym. J..

[50] Fridrihsone-Girone A., Stirna U., Misane M., Lazdiņa B., Deme L. (2016). Spray-applied 100% volatile organic compounds free two component polyurethane coatings based on rapeseed oil polyols. Prog. Org. Coat..

[51] Considine D.M., Considine G.D. (1995). Van Nostrand’s Scientific Encyclopedia.

[52] Kurańska M., Prociak A. (2016). The influence of rapeseed oil-based polyols on the foaming process of rigid polyurethane foams. Ind. Crops Prod..

[53] Zieleniewska M., Leszczyński M.K., Kurańska M., Prociak A., Szczepkowski L., Krzyżowska M., Ryszkowska J. (2015). Preparation and characterisation of rigid polyurethane foams using a rapeseed oil-based polyol. Ind. Crops Prod..

[54] Kirpluks M., Vanags E., Abolins A., Michalowski S., Fridrihsone A., Cabulis U. (2020). High Functionality Bio-Polyols from Tall Oil and Rigid Polyurethane Foams Formulated Solely Using Bio-Polyols. Materials.

[55] Kirpluks M., Pomilovskis R., Vanags E., Abolins A., Mierina I., Fridrihsone A. (2020). Optimisation of the chemo-enzymatic epoxidation of tall oil fatty acids using response surface methodology. J. Clean. Prod..

[56] Hawkins M.C., O’Toole B., Jackovich D. (2005). Cell Morphology and Mechanical Properties of Rigid Polyurethane Foam. J. Cell. Plast..

[57] Szycher M. (1999). Szycher’s Handbook of Polyurethanes.

